# Chromosome assembled and annotated genome sequence of *Aspergillus flavus* NRRL 3357

**DOI:** 10.1093/g3journal/jkab213

**Published:** 2021-06-26

**Authors:** Jeffrey M Skerker, Kaila M Pianalto, Stephen J Mondo, Kunlong Yang, Adam P Arkin, Nancy P Keller, Igor V Grigoriev, N Louise Louise Glass

**Affiliations:** 1 Innovative Genomics Institute, University of California, Berkeley, Berkeley, CA 94720, USA; 2 The U.S. Department of Energy Joint Genome Institute, Lawrence Berkeley National Laboratory, Berkeley, CA 94720, USA; 3 Department of Agricultural Biology, Colorado State University, Fort Collins, CO 80523, USA; 4 Department of Medical Microbiology & Immunology and Bacteriology, University of Wisconsin, Madison, WI 53706, USA; 5 Department of Bioengineering, University of California, Berkeley, Berkeley, CA 94720, USA; 6 Department of Plant and Microbial Biology, University of California, Berkeley, Berkeley, CA 94720, USA

**Keywords:** *Aspergillus flavus*, NRRL 3357, PacBio, Nanopore, genome sequence

## Abstract

*Aspergillus flavus* is an opportunistic pathogen of crops, including peanuts and maize, and is the second leading cause of aspergillosis in immunocompromised patients. *A. flavus* is also a major producer of the mycotoxin, aflatoxin, a potent carcinogen, which results in significant crop losses annually. The *A. flavus* isolate NRRL 3357 was originally isolated from peanut and has been used as a model organism for understanding the regulation and production of secondary metabolites, such as aflatoxin. A draft genome of NRRL 3357 was previously constructed, enabling the development of molecular tools and for understanding population biology of this particular species. Here, we describe an updated, near complete, telomere-to-telomere assembly and re-annotation of the eight chromosomes of *A. flavus* NRRL 3357 genome, accomplished via long-read PacBio and Oxford Nanopore technologies combined with Illumina short-read sequencing. A total of 13,715 protein-coding genes were predicted. Using RNA-seq data, a significant improvement was achieved in predicted 5’ and 3’ untranslated regions, which were incorporated into the new gene models.

## Introduction

*Aspergillus flavus* is an opportunistic plant pathogen, human pathogen, and a saprophyte. Agriculturally, *A. flavus* colonizes crops such as maize, peanuts, and cotton, both pre- and post-harvest (Klich 2007). While colonizing these crops, *A. flavus* produces aflatoxin, which is both toxic to mammals and a potent carcinogen (Williams *et al.* 2004; Liu and Wu 2010; Wild and Gong 2010), chronically impacting an estimated 4.5 billion people (CDC 2016). In the United States, mycotoxin production causes estimated yearly agricultural losses in corn ranging from tens of millions to over $1 billion (Mitchell *et al.* 2016). In addition to mycotoxin production, *A. flavus* is also a leading cause of invasive aspergillosis in humans, as well as the leading cause of fungal sinusitis and keratitis in tropical climates (Krishnan *et al.* 2009; Pasqualotto 2009; Rudramurthy *et al.* 2019).

The *A. flavus* isolate NRRL 3357 produces high levels of aflatoxin and has been developed as a model for the development of molecular tools and for dissecting the regulation and production of aflatoxin (Georgianna and Payne 2009; Amaike and Keller 2011). Previously, the genome sequence of *A. flavus* NRRL 3357 was sequenced using the whole-genome shotgun method (Nierman *et al.* 2015) and assembled into 958 contigs comprising 331 scaffolds. To enhance genome-wide studies in *A. flavus* for both functional and population genomics studies, we sought to generate a more complete genome assembly and annotation. Here, we report an updated, near complete, telomere-to-telomere assembly of the *A. flavus* strain NRRL 3357 genome, with 8 scaffolds corresponding to the 8 chromosomes of this species. Genome annotation, assisted by publicly available RNA-seq data, yielded greater resolution of 5’ and 3’ UTRs in this organism, with nearly half of genes containing annotated UTRs. We manually curated over 200 previously published genes, verifying that new predicted gene models corresponded with prior gene models and RNA-seq datasets.

## Materials and methods

### Fungal strain culture and DNA extraction

*A. flavus* NRRL 3357 was originally isolated from peanut, with our sample originating in the Keller lab strain collection (Hesseltine *et al.* 1966). Conidia from a week-old culture were grown in glucose minimal medium + 0.5% yeast extract at 30°C overnight. Genomic DNA isolation for genome sequencing of *A. flavus* was previously reported (Drott *et al.* 2020). Briefly, powdered lyophilized mycelia were resuspended in LETS buffer (20 mM EDTA pH 8.0, 0.5% SDS, 10 mM Tris-HCl pH 8.0, and 0.1 M LiCl). Genomic DNA was extracted using phenol: chloroform: isoamyl alcohol (25:24:1), followed by ethanol precipitation. gDNA was collected either by spooling or centrifugation, then washed with 70% ethanol and allowed to air dry. gDNA was resuspended in 10 mM Tris-HCl (pH 8.0) + 3.33 μg/mL RNase A and heated at 65°C for 30 minutes.

### Sequencing

DNA quality control, library preparation for PacBio and Illumina, and sequencing were performed at the Vincent Coates Genomics Sequencing Laboratory at the University of California, Berkeley. For the PacBio sequencing, DNA quantification and quality control were performed using a Femto Pulse System (Agilent Technologies), and high molecular weight DNA (∼130 kbp average size) was used to construct BluePippin (Sage Science) size-selected (>30-kbp) SMRTbell libraries (PacBio). PacBio libraries were sequenced on the Sequel Platform using the S/P2-C2 polymerase and version 5.0 chemistry on four Sequel SMRT cells, generating a total of ∼1.8 M reads with average read length of 13,122 bp. For Illumina sequencing, small (∼540 bp) insert libraries were generated using KAPA DNA HyperPrep kit with PCR-free protocol (Roche), generating ∼319 M PE150 reads. Oxford Nanopore libraries were generated using ∼15 μg of high molecular weight DNA and the SQK-LSK308 kit. Oxford Nanopore sequencing was performed in-house using the minION platform and a combination of live base calling using minKNOW App (v1.11.5) and offline base calling using Albacore App (v2.3.3) (Oxford Nanopore). Three FLO-MIN107 flow cells (version 9.5.1 pore chemistry) were used to generate ∼1.4 M reads with average length of 3050 bp. The assembled genome sequence has been used to assess population genomics of *A. flavus* (Drott *et al.*^2020^, ^2021^).

### Genome assembly and annotation

The combined PacBio and Oxford Nanopore long-read datasets were used to generate a hybrid *de novo* assembly using the CANU assembler (v.1.7.1) (Koren *et al.* 2017) with default settings, except the genome size was set to 40 Mbp and stopOnReadQuality set to “false.” The final read depth coverage, after filtering for reads <1 kbp, was ∼700X. The CANU scaffolds were polished using the PacBio Sequel data and a combination of pbalign (v.0.3.1), blasr (v.5.3), and arrow (v.2.2.2) from the SMRT Link package (v.5.1.0.26412, PacBio). A final error correction step was performed using the Illumina data (∼650X coverage) with a combination of bwa (v.0.7.17) (Li 2013), samtools (v.1.9) (Li *et al.* 2009), and pilon (v.1.22) (Walker *et al.* 2014). For the most accurate final assembly of the *A. flavus* genome, Illumina data and at least one round of pilon correction were needed. For the discovery of *de novo* repeats Repeatscout v1.0.5 (Price *et al.* 2005) was used and for repeat masking, RepBase (Bao *et al.* 2015) and RepeatMasker (Smit *et al.* 1996-2010) were used. 

Annotation of the genome was performed using the Joint Genome Institute (JGI) Genome Annotation pipeline (Grigoriev *et al.* 2014) using publicly available RNA-seq data (SRA datasets: SRR2632952, SRR2632961, SRR2632962, SRR2632963, SRR2632966, SRR2633059, SRR2633060, SRR2633061, SRR2633139, SRR5061895, SRR5061899, SRR5061903, SRR5061905, SRR5061908, SRR5061909, SRR544871, SRR544872, SRR544873, SRR8115610, SRR8115611, SRR8115612, SRR8115613, SRR8115614, and SRR8115615). Previously produced gene models available from FungiDB (Stajich *et al.* 2012) were mapped forward to the new assembly. The final annotation included 13,715 gene models, of which 43.29% represent previously produced models mapped forward from FungiDB (fungidb.org/), while the remainder of gene models were updated/improved based on transcriptomics data and the improved assembly. For predicted short genes (*i.e.*, <200AA), we evaluated predicted annotations including signal peptides, transmembrane domains, InterPro domain annotations, or support through self-clustering. If any of these types of support were detected, short gene models were retained in our final gene set. In addition, although alternative splice forms were not included in our final gene catalog, RNA-seq based models from tools like COMBEST (Zhou *et al.* 2015) are available as tracks on the Aspfl2_3 genome browser https://mycocosm.jgi.doe.gov/Aspfl2_3 and enable reconstruction of alternative splice forms.

### Data availability

The whole-genome assembly and annotation and the *A. flavus* mitochondrial DNA sequence are available from the JGI MycoCosm portal (Grigoriev *et al.* 2014) at https://mycocosm.jgi.doe.gov/Aspfl2_3 and have been deposited at GenBank under accession numbers CP044616-CP044623, Bioproject accession number PRJNA575750. Raw sequencing reads have been deposited under SRA project accession number PRJNA637788. Manually curated genes and references are included in Supplementary Table S1. Supplementary material is available at figshare: https://doi.org/10.25387/g3.14738154.

## Results and discussion

**Figure 1:**
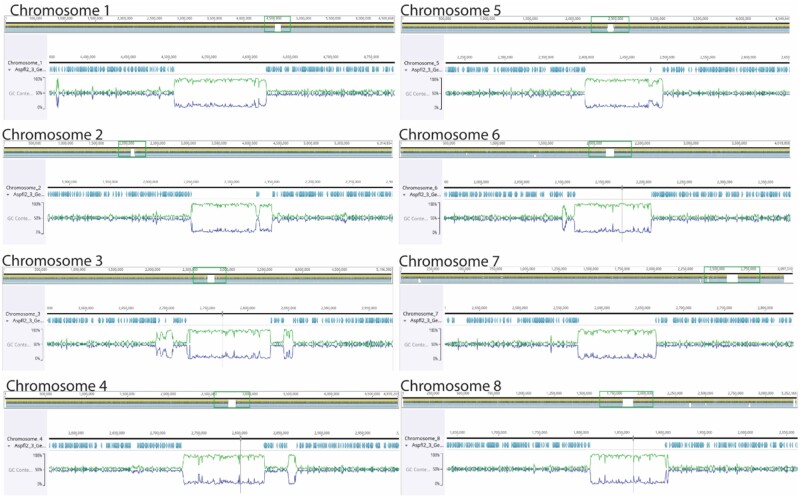
Graphic representation of the 8 NRRL 3357 *Aspergillus flavus* assembled chromosomes. Green traces represent AT content and dark blue traces represent GC content. Teal-colored arrows indicate predicted coding regions^1^. ^1^Figure generated using Geneious Prime v.2021.0.3; www.geneiousprime.com.

The final genome assembly of NRRL 3357 resulted from a combined long- (PacBio SMRT and Oxford Nanopore) and short-read (Illumina) sequencing methods was 37.75 Mbp in 8 contigs, a significant improvement from the previous assembly which contained 331 scaffolds (Table 1) (Nierman *et al.* 2015) and a genome size slightly less the 37 Mbp. The industrially relevant species, *Aspergillus oryzae*, is closely related to *A. flavus* and *A. flavus* chromosome names are based on the *A. oryzae* genome (Machida *et al.* 2005; Figure 1). The final sequencing read depth was 650X, and the average GC content across the genome was 47.34%. Seven of eight chromosomes are represented by complete telomere-to-telomere assemblies. It was not possible to complete the assembly of the right end of chromosome 7 due to the presence of a large rDNA repeat. The new assembly increased in size relative to the original assembly (Nierman *et al.* 2015) primarily due to the improved assembly of repetitive regions other than the rDNA region, resulting in an increase from 1.1% of the genome to 3.47% (Table 1), including a significant increase in Mariner Tc1 repeats [43% in previous assembly (Nierman *et al.* 2015) compared to 83.18% in the current assembly]. We also identified 15 out of 16 telomeres as well as predicted centromeric regions (Figure 1). Each chromosome was flanked by 10–13 telomeric repeats “TTAGGGTCAACA” that were identical to those identified in the *A. oryzae* (Kusumoto *et al.* 2003). In filamentous fungi, centromeric regions have high AT content, are ∼100 kb and typically lack coding regions (Smith *et al.* 2012). In the assembled *A. flavus* genome, predicted centromeric regions were identified using these criteria and were ∼100 kbp, but some chromosomes had additional AT-rich regions surrounding predicted centromeric regions (Figure 1). Verification of functional centromeric regions of the *A. flavus* genome would require further experimentation, for example, the identification of the specialized histone H3 variant [called CenH3 in *Neurospora crassa*) (Smith *et al.* 2011)], a so-called universal “centromere identifier.” In our new assembly, the mitochondrial DNA was identical to that previously published (Joardar *et al.* 2012).

**Table 1:** Summary of assembly and annotation statistics of the *A. flavus* NRRL 3357 genome

Assembly statistics	**Illumina assembly (Nierman** 2015 **)^*a*^**	**Illumina assembly, reannotated (Hatmaker** 2020 **)^*b*^**	Mixed sequencing assembly (this work)
Genome size (Mbp)	36.89	36.89	37.75
Coverage	5X	5X	650X
Number of scaffolds/contigs	958/331	958/331	8
L50	6	6	4
N50, Mbp (Scaffold)	2.39	2.39	4.81
Complete chromosomes	—	—	8
% genes by BUSCO assessment^*c*^: Fungi set			
Single-copy	92.70	96.30	98.70
Duplicated	0.30	1.70	0.50
Fragmented	3.00	0.90	0.00
Annotation statistics			
Number of predicted protein coding genes	13,485	14,313	13,715
Percent gene models with annotated UTRs	9.25%	—	44.43%
Predicted secondary metabolite clusters	56		83^*d*^
Predicted CAZymes	627		644
Total repeat length (bp)	404,315 (1.1%)		1,311,342 (3.47%)

a Nierman *et al.* (2015).

b Hatmaker *et al.* (2020).

c Seppey *et al.* (2019).

d Drott *et al.* (2021).

Our final annotation contained 13,715 predicted protein coding genes, of which 43.29% of the final filtered gene model set represent previously produced models mapped forward from FungiDB (https://fungidb.org/). The remaining gene models were updated and improved based on the improved assembly and on publicly available transcriptomics data (see Materials and Methods). This gene model count was slightly lower than a recently updated, transcriptome-based annotation of the 331 scaffolds of the *A. flavus* 2015 assembly (Nierman *et al.* 2015), which relied on RNA isolated from *A. flavus* in six conditions and used a combination of different *ab initio* gene predictors (Hatmaker *et al.* 2020). A BUSCO v5.0 (Seppey *et al.* 2019) analyses, calculated using the Fungi dataset, revealed that 98.7% of BUSCO genes were captured in single copy in our new assembly and annotation, *vs* 96.3% in the Hatmaker *et al.* (2020) and 92.7% from Nierman *et al.* (2015) versions (Table 1). In addition, fragmented and duplicated genes were less frequent in our version relative to other annotations. The total number of gene models in our version (13,715 genes) and the Nierman *et al.*, annotation (13,485 genes) (Nierman *et al.* 2015) was slightly lower than gene models predicted by Hatmaker *et al.*, (14,313 genes) (Hatmaker *et al.* 2020; Table 1). This discrepancy may partially result from our filtering methods, which aimed to remove transposable elements and short, unsupported gene models from the final gene catalog. We also used publicly available transcriptomic data to improve the annotation of 5’ and 3’ UTRs on our filtered gene models, which were absent or rarely predicted in other models. We UTRs for 44.43% of our models (6093 genes), a significant increase from the 9.25% UTRs identified from previous studies.

To validate updated annotation methods using the mapped-forward gene models, we chose a set of 172 genes that had been described in the literature to manually curate and to compare our new JGI models with FungiDB gene models (Supplementary Table S1). Within this set, we used publicly available RNA-seq data to provide 5’ and 3’ untranslated regions (UTRs) information and homology modeling to confirm gene models. In 68/172 genes, the models did not change, while for an additional 47 genes, the gene predictions were 100% identical, but UTRs were added (Supplementary Table S1). In 57 cases, the existing FungiDB gene model was corrected using newly available RNAseq and homology evidence. For a few gene models, it was difficult to define the correct model, primarily due to the absence of RNA-seq data or differences in predicted gene models when orthologs in other species were evaluated. In the Mycocosm portal (https://mycocosm.jgi.doe.gov/Aspfl2_3/Aspfl2_3.home.html), we provide a link to the closest FungiDB AFLA model for users to compare the new models with the previous annotation. Overall, our mapped-forward gene models, using the complete genome assembly and RNA-seq data provided more complete gene models for *A. flavus* and with significantly more 5’ and 3’ UTR information compared to previous versions.

As *A. flavus* is a plant pathogen and saprophyte, carbohydrate active enzymes (CAZymes) are important for this fungus’s ability to grow on plant material. With our updated annotation, we observed a slight increase in the number of predicted CAZymes, from 627 to 644, in the updated *A. flavus* genome annotation as predicted by CAZy Database (Lombard *et al.* 2014). *A. flavus* also produces a wealth of bioactive secondary metabolites from biosynthetic gene clusters (BGCs); the function and structure of these secondary metabolites is an active area of research with this organism (Greco *et al.* 2019; Keller 2019). Through prior studies, 56 secondary metabolite clusters have been identified in the *A. flavus* genome (Georgianna *et al.* 2010; Marui *et al.* 2011; Amare and Keller 2014; Nierman *et al.* 2015). Using this current *A. flavus* genome assembly for NRRL 3357, coupled with assessing BGC diversity in *A. flavus* populations, 83 BCGs were identified in the *A. flavus* NRRL 3357 genome (Drott *et al.* 2021). The genomic positions for these 83 BCGs in our assembled *A. flavus* NRRL 3357 genome are available as supplemental data from Drott *et al.* (2021).

In summary, we provide an improved, near-complete, telomere-to-telomere genome assembly for *A. flavus* NRRL 3357, resulting from combined Illumina, Oxford Nanopore, and PacBio SMRT sequencing data. This updated assembly has been useful for assessing population genomics of 94 isolates of *A. flavus* isolated from different geographic locations in the United States (Drott *et al.* 2020) and BCG diversity (Drott *et al.* 2021). The genome assembly consists of 8 contigs representing 8 chromosomes, with 15 of 16 telomeric repeats and all centromeric sequences identified and assembled. The total genome assembly size is 37.75 Mbp, and the updated annotation, supported by RNA-seq and homology data, yielded 13,715 predicted protein-coding gene models.

## Funding

Funding for this project was provided by a grant to N.L.G., J.M.S., and A.P.A. through the Innovative Genomics Institute at University of California, Berkeley. Genome annotation performed by the Joint Genome Institute, a Department of Energy (DOE) Office of Science User Facility, was supported by the Office of Science of the US DOE under Contract no. DE-AC02-05CH11231. 

## Conflicts of interest

None declared. 

## References

[jkab213-B1] Amaike S , Keller NP. 2011. Aspergillus flavus. Annu Rev Phytopathol. 49:107–133. 21513456 10.1146/annurev-phyto-072910-095221

[jkab213-B2] Amare MG , Keller NP. 2014. Molecular mechanisms of *Aspergillus flavus* secondary metabolism and development. Fungal Genet Biol. 66:11–18. 24613992 10.1016/j.fgb.2014.02.008

[jkab213-B3] Bao W , Kojima KK, Kohany O. 2015. Repbase Update, a database of repetitive elements in eukaryotic genomes. Mob DNA. 6: 11. 26045719 10.1186/s13100-015-0041-9 PMC4455052

[jkab213-B4] CDC 2016. Health studies. Understanding chemical exposures. Aflatoxin. CDC, Atlanta, GA: http://www.cdc.gov/nceh/hsb/chemicals/aflatoxin.htm.

[jkab213-B5] Drott MT , Rush TA, Satterlee TR, Giannone RJ, Abraham PE, et al 2021. Microevolution in the pan-secondary metabolome of *Aspergillus flavus* has macroevolutionary implications for filamentous fungi. Proc Natl Acad Sci USA. 118:e2021683118. 34016748 10.1073/pnas.2021683118 PMC8166093

[jkab213-B6] Drott MT , Satterlee TR, Skerker JM, Pfannenstiel BT, Glass NL, et al 2020. The frequency of sex: population genomics reveals differences in recombination and population structure of the aflatoxin-producing fungus *Aspergillus flavus*. mBio. 11:e00963-20. 32665272 10.1128/mBio.00963-20 PMC7360929

[jkab213-B7] Georgianna DR , Fedorova ND, Burroughs JL, Dolezal AL, Bok JW, et al 2010. Beyond aflatoxin: four distinct expression patterns and functional roles associated with *Aspergillus flavus* secondary metabolism gene clusters. Mol Plant Pathol. 11:213–226. 20447271 10.1111/j.1364-3703.2009.00594.x PMC4116135

[jkab213-B8] Georgianna DR , Payne GA. 2009. Genetic regulation of aflatoxin biosynthesis: from gene to genome. Fungal Genet Biol. 46:113–125. 19010433 10.1016/j.fgb.2008.10.011

[jkab213-B9] Greco C , Keller NP, Rokas A. 2019. Unearthing fungal chemodiversity and prospects for drug discovery. Curr Opin Microbiol. 51:22–29. 31071615 10.1016/j.mib.2019.03.002 PMC6832774

[jkab213-B10] Grigoriev IV , Nikitin R, Haridas S, Kuo A, Ohm R, et al 2014. MycoCosm portal: gearing up for 1000 fungal genomes. Nucleic Acids Res. 42:D699–704. 24297253 10.1093/nar/gkt1183 PMC3965089

[jkab213-B11] Hatmaker EA , Zhou X, Mead ME, Moon H, Yu JH, et al 2020. Revised transcriptome-based gene annotation for *Aspergillus flavus* Strain NRRL 3357. Microbiol Resour Announc. 9:e01155-20. 33273000 10.1128/MRA.01155-20 PMC7714855

[jkab213-B12] Hesseltine CW , Shotwell OL, Ellis JJ, Stubblefield RD. 1966. Aflatoxin formation by *Aspergillus flavus*. Bacteriol Rev. 30:795–805. 5342522 10.1128/br.30.4.795-805.1966 PMC441016

[jkab213-B13] Joardar V , Abrams NF, Hostetler J, Paukstelis PJ, Pakala S, et al 2012. Sequencing of mitochondrial genomes of nine Aspergillus and Penicillium species identifies mobile introns and accessory genes as main sources of genome size variability. BMC Genomics. 13:698. 23234273 10.1186/1471-2164-13-698 PMC3562157

[jkab213-B14] Keller NP. 2019. Fungal secondary metabolism: regulation, function and drug discovery. Nat Rev Microbiol. 17:167–180. 30531948 10.1038/s41579-018-0121-1 PMC6381595

[jkab213-B15] Klich MA. 2007. *Aspergillus flavus*: the major producer of aflatoxin. Mol Plant Pathol. 8:713–722. 20507532 10.1111/j.1364-3703.2007.00436.x

[jkab213-B16] Koren S , Walenz BP, Berlin K, Miller JR, Bergman NH, et al 2017. Canu: scalable and accurate long-read assembly via adaptive k-mer weighting and repeat separation. Genome Res. 27:722–736. 28298431 10.1101/gr.215087.116 PMC5411767

[jkab213-B17] Krishnan S , Manavathu EK, Chandrasekar PH. 2009. *Aspergillus flavus*: an emerging non-fumigatus Aspergillus species of significance. Mycoses. 52:206–222. 19207851 10.1111/j.1439-0507.2008.01642.x

[jkab213-B18] Kusumoto KI , Suzuki S, Kashiwagi Y. 2003. Telomeric repeat sequence of *Aspergillus oryzae* consists of dodeca-nucleotides. Appl Microbiol Biotechnol. 61:247–251. 12698283 10.1007/s00253-002-1193-3

[jkab213-B19] Li H , Handsaker B, Wysoker A, Fennell T, Ruan J, et al; 1000 Genome Project Data Processing Subgroup. 2009. The sequence alignment/map format and SAMtools. Bioinformatics. 25:2078–2079. 19505943 10.1093/bioinformatics/btp352 PMC2723002

[jkab213-B20] Liu Y , Wu F. 2010. Global burden of aflatoxin-induced hepatocellular carcinoma: a risk assessment. Environ Health Perspect. 118:818–824. 20172840 10.1289/ehp.0901388 PMC2898859

[jkab213-B38] Li H. (2013). Aligning sequence reads, clone sequences and assembly contigs with BWA-MEM. arXiv:1303.3997v2.

[jkab213-B21] Lombard V , Golaconda Ramulu H, Drula E, Coutinho PM, Henrissat B. 2014. The carbohydrate-active enzymes database (CAZy) in 2013. Nucleic Acids Res. 42:D490–D495. 24270786 10.1093/nar/gkt1178 PMC3965031

[jkab213-B22] Machida M , Asai K, Sano M, Tanaka T, Kumagai T, et al 2005. Genome sequencing and analysis of *Aspergillus oryzae*. Nature. 438:1157–1161. 16372010 10.1038/nature04300

[jkab213-B23] Marui J , Yamane N, Ohashi-Kunihiro S, Ando T, Terabayashi Y, et al 2011. Kojic acid biosynthesis in *Aspergillus oryzae* is regulated by a Zn(II)(2)Cys(6) transcriptional activator and induced by kojic acid at the transcriptional level. J Biosci Bioeng. 112:40–43. 21514215 10.1016/j.jbiosc.2011.03.010

[jkab213-B24] Mitchell NJ , Bowers E, Hurburgh C, Wu F. 2016. Potential economic losses to the US corn industry from aflatoxin contamination. Food Addit Contam Part A Chem Anal Control Expo Risk Assess. 33:540–550. 26807606 10.1080/19440049.2016.1138545 PMC4815912

[jkab213-B25] Nierman WC , Yu J, Fedorova-Abrams ND, Losada L, Cleveland TE, et al 2015. Genome sequence of *Aspergillus flavus* NRRL 3357, a strain that causes aflatoxin contamination of food and feed. Genome Announc. 3:e00168-15. 25883274 10.1128/genomeA.00168-15 PMC4400417

[jkab213-B26] Pasqualotto AC. 2009. Differences in pathogenicity and clinical syndromes due to *Aspergillus fumigatus* and *Aspergillus flavus*. Med Mycol. 47(Suppl.):S261–S270. 18654921 10.1080/13693780802247702

[jkab213-B27] Price AL , Jones NC, Pevzner PA. 2005. *De novo* identification of repeat families in large genomes. Bioinformatics. 21(Suppl.):i351–i358. 15961478 10.1093/bioinformatics/bti1018

[jkab213-B28] Rudramurthy SM , Paul RA, Chakrabarti A, Mouton JW, Meis JF. 2019. Invasive Aspergillosis by *Aspergillus flavus*: epidemiology, diagnosis, antifungal Resistance, and management. J Fungi (Basel). 5:55. 10.3390/jof5030055 PMC6787648 31266196

[jkab213-B29] Seppey M , Manni M, Zdobnov EM. 2019. 2019 BUSCO: assessing genome assembly and annotation completeness. Methods Mol Biol. 1962:227–245. 31020564 10.1007/978-1-4939-9173-0_14

[jkab213-B30] Smit AFA , Hubley R, Green P. 1996-2010. RepeatMasker Open-3.0. http://www.repeatmasker.org.

[jkab213-B31] Smith KM , Galazka JM, Phatale PA, Connolly LR, Freitag M. 2012. Centromeres of filamentous fungi. Chromosome Res. 20:635–656. 22752455 10.1007/s10577-012-9290-3 PMC3409310

[jkab213-B32] Smith KM , Phatale PA, Sullivan CM, Pomraning KR, Freitag M. 2011. Heterochromatin is required for normal distribution of *Neurospora crassa* CenH3. Mol Cell Biol. 31:2528–2542. 21505064 10.1128/MCB.01285-10 PMC3133421

[jkab213-B33] Stajich JE , Harris T, Brunk BP, Brestelli J, Fischer S, et al 2012. FungiDB: an integrated functional genomics database for fungi. Nucleic Acids Res. 40:D675–D681. 22064857 10.1093/nar/gkr918 PMC3245123

[jkab213-B34] Walker BJ , Abeel T, Shea T, Priest M, Abouelliel A, et al 2014. Pilon: an integrated tool for comprehensive microbial variant detection and genome assembly improvement. PLoS One. 9:e112963. 25409509 10.1371/journal.pone.0112963 PMC4237348

[jkab213-B35] Wild CP , Gong YY. 2010. Mycotoxins and human disease: a largely ignored global health issue. Carcinogenesis. 31:71–82. 19875698 10.1093/carcin/bgp264 PMC2802673

[jkab213-B36] Williams JH , Phillips TD, Jolly PE, Stiles JK, Jolly CM, et al 2004. Human aflatoxicosis in developing countries: a breview of toxicology, exposure, potential health consequences, and interventions. Am J Clin Nutr. 80:1106–1122. 15531656 10.1093/ajcn/80.5.1106

[jkab213-B37] Zhou K , Salamov A, Kuo A, Aerts AL, Kong X, et al 2015. Alternative splicing acting as a bridge in evolution. Stem Cell Investig. 2:19. 10.3978/j.issn.2306-9759.2015.10.01 PMC4923640 27358887

